# Polyisobutylene Urolithiasis Due to Ileal Conduit Urostomy Appliance: An Index Case

**DOI:** 10.1089/cren.2015.29016.maa

**Published:** 2015-10-01

**Authors:** Michael A. Avallone, Ann M. Kolbach-Mandel, Ian C. Mandel, Neil S. Mandel, Peter N. Dietrich, Jeffrey A. Wesson, Carley M. Davis

**Affiliations:** ^1^ Department of Urology, Medical College of Wisconsin, Milwaukee, Wisconsin.; ^2^ Mandel International Stone and Molecular Analysis Center, Milwaukee, Wisconsin.; ^3^ Division of Nephrology, Department of Medicine, Medical College of Wisconsin, Milwaukee, Wisconsin.; ^4^ Clement J. Zablocki Department of Veterans Affairs Medical Center, Milwaukee, Wisconsin.

## Abstract

Polyisobutylene (PIB) is a synthetic elastomer that is a component of sealants, adhesives, and chewing gum base. We report a case of bilateral PIB urolithiasis in a patient with an ileal conduit urinary diversion due to neurogenic bladder from spinal cord injury. Infrared spectroscopy confirmed the composition of bilateral stones and adhesive from the patient's urostomy appliance to be PIB. No previous cases of PIB urolithiasis are reported in the literature.

## Clinical History

The patient is a 65-year-old female with a history of T7 spinal cord injury in 1964 resulting in complete paraplegia and neurogenic bladder. Approximately 50 years ago, the patient underwent simple cystectomy and ileal conduit urinary diversion. She has subsequently undergone multiple procedures for recurrent bilateral nephrolithiasis. Previous stone analyses demonstrated calcium phosphate and magnesium ammonium phosphate calculi. More recently, a stone analysis performed by our hospital's affiliated clinical pathology service was inconclusive, reporting “extraneous material embedded with many calcium oxalate and calcium phosphate crystals.” A diagnosis of hyperparathyroidism was made, but the patient has refused parathyroidectomy due to fear of anesthesia.

In early 2015, the patient presented with fever, tachycardia, emesis, and abdominal pain. On evaluation, she was found to have multiple bilateral obstructing ureteral stones. Remarkably, the patient was at 4 months status post ureteroscopy and Holmium laser lithotripsy with postoperative imaging confirming the absence of residual stone. She emergently underwent endoscopy per ileal conduit with bilateral ureteral stent placement. The sepsis resolved and she returned for elective management of the bilateral ureteral stones.

## Physical Examination

At the time of outpatient follow-up, the patient's physical examination was noteworthy for a patent ileal conduit stoma productive of clear yellow urine and a stable neurologic examination with absent sensation inferior to the costal margin.

## Diagnostic Studies

The urine culture from the time of presentation with sepsis grew *Proteus mirabilis* and subsequent preoperative urine cultures from the ileal conduit grew multiple organisms, including *Enterococcus faecalis*, Corynebacterium species, and *Staphylococcus epidermidis*. Urinalysis at that time was noteworthy for a pH of 7.0, 1+ proteinuria, microscopic hematuria, pyuria, and bacteria. The results from two consecutive Litholink^®^ (Litholink Corporation, Chicago, IL) 24-hour urine analyses are reported in Table 1. The remainder of her laboratory evaluation was remarkable for diminished renal function (calculated creatinine clearance of 63 mL/min), hypercalcemia (11.1 mg/dL, normal range 8.6–10.2), and an elevated intact parathyroid hormone (182 pg/mL, normal range 15.0–72.0).

**Table 1.** Twenty-Four Hour Urine Analysis

*Component*	*Collection 1*	*Collection 2*	*Reference range*
Urine volume (L/day)	0.8	1.1	0.5–4.0
Supersaturation calcium oxalate	3	3.3	6.0–10.0
Calcium (mg/day)	77	103	0–199
Oxalate (mg/day)	19	27	20–40
Citrate (mg/day)	161	99	≥551
Supersaturation calcium phosphate	0.9	0.8	0.5–2.0
pH	9.1	9.1	5.8–6.2
Supersaturation uric acid	0.0	0.0	0.0–1.0
Uric acid (g/day)	0.2	0.3	0.0–0.75
Creatinine (mg/day)	485	476	
Sodium (mmol/day)	62	91	50–150
Potassium (mmol/day)	35	27	20–100
Magnesium (mg/day)	7	6	30–120
Phosphorous (g/day)	0.3	0.4	0.60–1.20
Patient weight (kg)	74.8	74.8	
Creatinine/kg (mg/kg)	6.5	6.4	15.0–20.0
Calcium/kg (mg/kg)	1.0	1.4	0.0–3.9
Calcium/creatinine (mg/g)	158	217	0–139
Ammonium (mmol/day)	178	264	15–60
Chloride (mEq/day)	63	93	70–250
Sulfate (mEq/day)	16	15	20–80
Urea nitrogen (g/day)	3.5	4.3	6.0–14.0
Protein catabolic rate (g/kg/day)	0.5	0.5	0.8–1.4
Cysteine screening	Negative	Negative	Negative

**FIG. 1.**
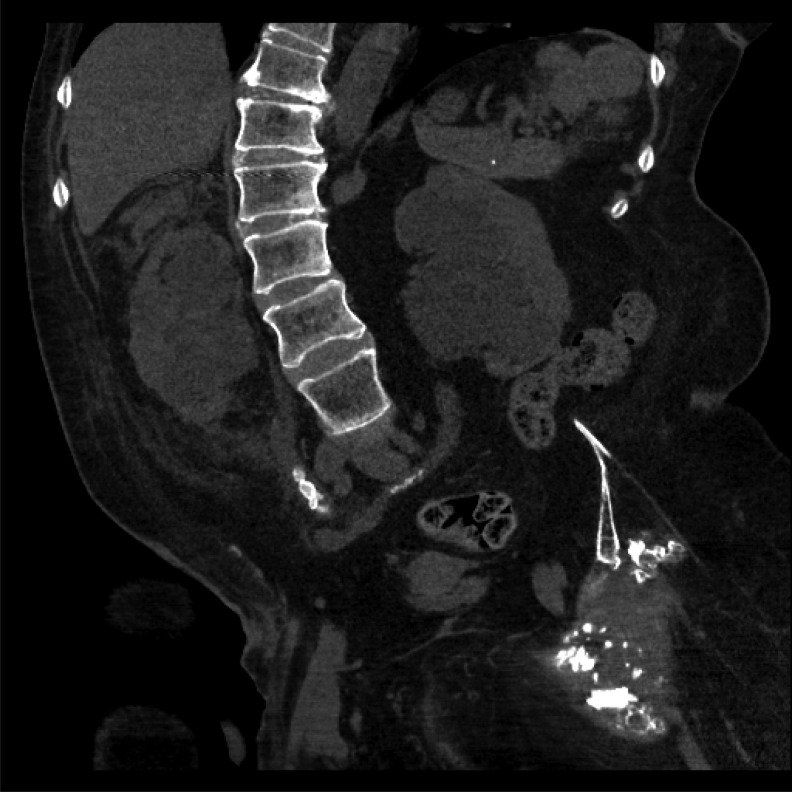
CT scan demonstrating bilateral hydroureteronephrosis, perinephric stranding and bilateral obstructing ureteral stones.

A CT noncontrast scan of the abdomen and pelvis demonstrated mildly atrophic kidneys with bilateral hydroureteronephrosis, perinephric fat stranding, and suggestion of right forniceal rupture (Fig. 1). A total of six stones ranging from 6 to 15 mm in size were obstructing the right mid-ureter, and four stones measuring 2 to 4 mm in size were present in the left mid-ureter. The mean density of the ureteral stones was 460 HU.

## Intervention

Treatment of the bilateral ureteral stone burden was undertaken in a staged manner. Left ureteroscopy was performed first through retrograde approach through the ileal conduit. The procedure was performed with the patient awake due to the patient's high neurologic level of injury and her anxiety regarding anesthesia. The ureteral stones were identified and Holmium laser lithotripsy was performed with basket extraction of the fragments. Lithotripsy of the stones was challenging due to the soft amorphous core of the stones with an adherent dense outer shell. The ureteral stones were removed, as well as several renal pelvis stones with the same unusual characteristics. One month later, the patient underwent right percutaneous nephrolithotomy and antegrade ureteroscopy to address the remaining contralateral stone burden. The stone consistency was again noted to be unusual with the same amorphous material composing the nucleus of the stones.

## Outcome

**FIG. 2.**
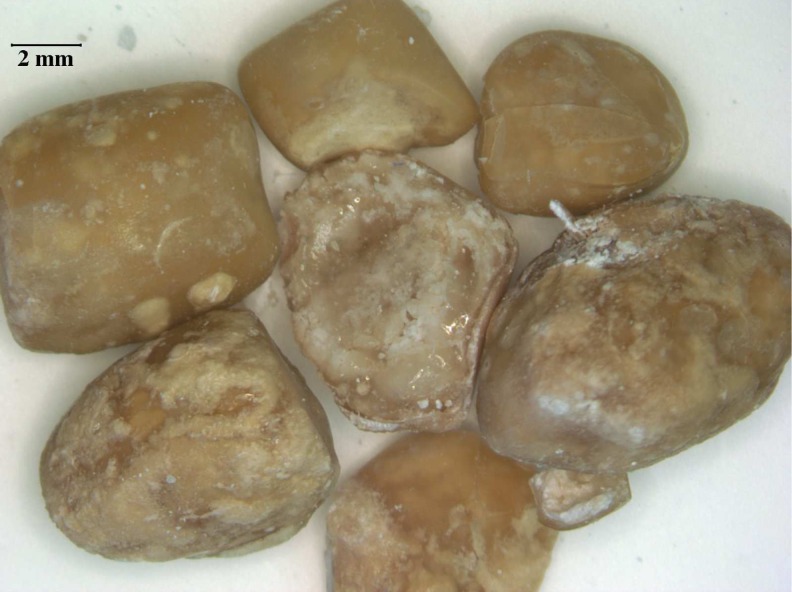
Gross appearance of the stones.

**FIG. 3.**
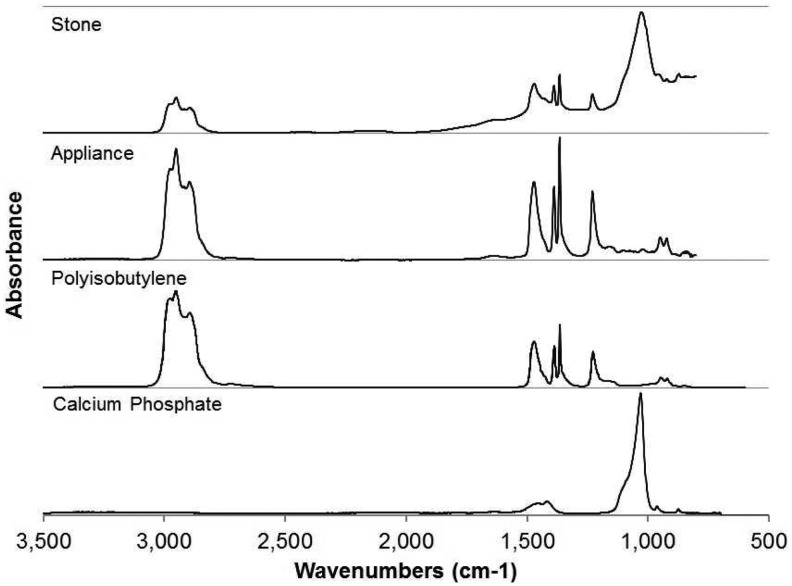
FTIR analysis results. FTIR, Fourier transform infrared spectroscopy.

The stones were analyzed at the Mandel International Stone and Molecular Analysis Center. The gross appearance of the intact stones is demonstrated in Figure 2. Compositional analysis was conducted by Fourier transform infrared spectroscopy (FTIR) using a Nicolet Nexus 870 FTIR Spectrometer (Thermo Fisher Scientific, Waltham, MA) running their proprietary operating software. Data were collected at room temperature using a diffuse transmission reflectance attachment with 32 scans per data collection between 700 and 3500 cm^−1^. Sample spectra were compared with reference spectra using a correlation analysis algorithm. Reference FTIR spectral libraries included a locally prepared and very comprehensive stone analysis library as well as a set of commercially available FTIR spectral libraries constructed by Sigma-Aldrich Chemical (Sigma-Aldrich, St. Louis, MO). The match of the infrared data for the patient's stones with the spectra for polyisobutylene (PIB) from the Aldrich Chemical HR FTIR Collection Series II (spectra #17943) was at the 96.1% level (Fig. 3). Additionally, a basic calcium phosphate crystal component was identified and appeared as a white powder within and on the surface of the stone sample.

**FIG. 4.**
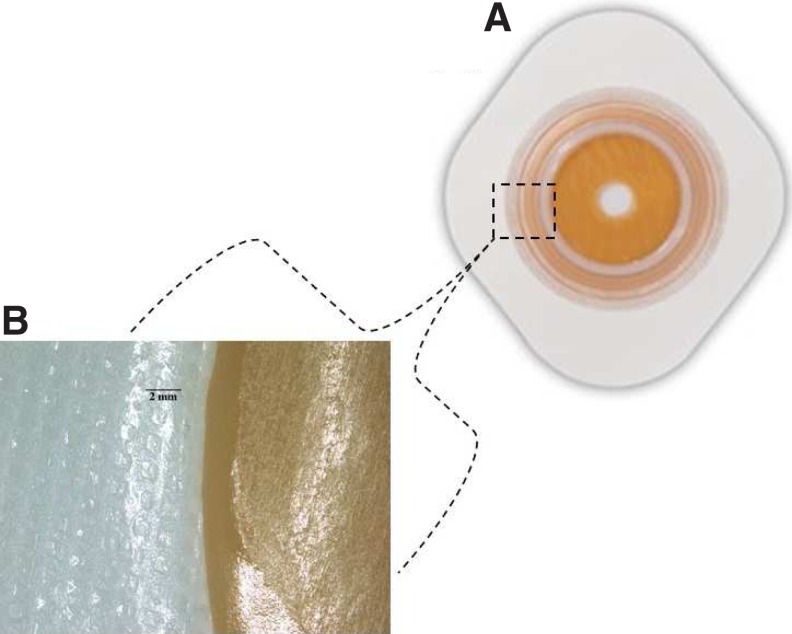
**(A)** ConvaTec appliance. **(B)** The surface in contact with the patient's skin that was confirmed with FTIR to contain polyisobutylene.

PIB (C_4_H_8_)_N_ is used in adhesives, sealing compounds, and is approved by Food and Drug Administration for use in chewing gum base.^1^ The patient denied frequent or excessive use of chewing gum. The medical application of PIB is primarily for the skin adhesives of transdermal drug delivery systems and ostomy appliances.^2^ Our patient did not use any transdermal medications, so we believed the source of PIB urolithiasis to be the ostomy appliance. The patient had been using a ConvaTec Natura^®^ (ConvaTec, Bridgewater, NJ) two-piece urostomy appliance. Two specific areas (Fig. 4) of the Natura^®^ ostomy appliance were characterized by FTIR and the composition of the adhesive side of the appliance matched with PIB (Fig. 4B). Since the adhesive of ostomy appliance was identified as the nidus for stone formation, the patient was provided an alternative type of urostomy appliance.

To the best of our knowledge, PIB urolithiasis has not previously been reported. PIB is an organic polymer with minimal, if any, solubility in water. Therefore, PIB would not be expected to enter the bloodstream by transdermal absorption for delivery to urine by filtration to generate stones. In this case, PIB from the adhesive on the wafer of the appliance most likely refluxed into the bilateral upper urinary tracts, creating a nidus for stone formation. A previous report of nephrolithiasis resulting from reflux of a staple from the proximal end of an ileal conduit supports this hypothesis.^3^ Crystal formation could then have ensued due to the patient's multiple risk factors, including hyperparathyroidism, low urine volume, and low urinary citrate associated with calcium stone formation, as well as high urine pH and chronic bacterial colonization associated with struvite lithogenesis. After formation, the stones would have been unable to traverse the ureteroenteric anastomosis, resulting in obstruction.

This report emphasizes the importance of using an analysis laboratory that goes beyond the standard operating procedures of most commercial laboratories. Commercial laboratories have been shown to accurately identify pure stones, but there is significant variability in the reporting of mixed and metabolic stones.^4^ The specialized analysis laboratory used in this study routinely discusses any unusual stone analysis results with the patient's physician who is often asked to provide a complete medication list along with insights into the patient's known diagnoses and overall health.

The present report discusses the index case of PIB urolithiasis resulting from the adhesive surface of the urostomy appliance for an ileal conduit. Our case emphasizes the importance of pursuing a more specialized stone analysis with infrared spectroscopy when unusual cases of urolithiasis are encountered.

## Abbreviations Used

CT: computed tomography
PIB: polyisobutylene
FTIR: Fourier transform infrared spectroscopy
